# Defining the Genetic Basis of Plant–Endophytic Bacteria Interactions

**DOI:** 10.3390/ijms20081947

**Published:** 2019-04-20

**Authors:** Artur Pinski, Alexander Betekhtin, Katarzyna Hupert-Kocurek, Luis A. J. Mur, Robert Hasterok

**Affiliations:** 1 Department of Plant Anatomy and Cytology, Faculty of Biology and Environmental Protection, University of Silesia in Katowice, 40-032 Katowice, Poland; alexander.betekhtin@us.edu.pl; 2 Department of Biochemistry, Faculty of Biology and Environmental Protection, University of Silesia in Katowice, 40-032 Katowice, Poland; katarzyna.hupert-kocurek@us.edu.pl; 3 Institute of Biological, Environmental and Rural Sciences, Aberystwyth University, Penglais Campus, Aberystwyth, Wales SY23 3DA, UK; lum@aber.ac.uk

**Keywords:** endophytic bacteria, plant interactions, colonisation, mutants, transcriptomics, metabolomics, proteomics, comparative genomics

## Abstract

Endophytic bacteria, which interact closely with their host, are an essential part of the plant microbiome. These interactions enhance plant tolerance to environmental changes as well as promote plant growth, thus they have become attractive targets for increasing crop production. Numerous studies have aimed to characterise how endophytic bacteria infect and colonise their hosts as well as conferring important traits to the plant. In this review, we summarise the current knowledge regarding endophytic colonisation and focus on the insights that have been obtained from the mutants of bacteria and plants as well as ‘omic analyses. These show how endophytic bacteria produce various molecules and have a range of activities related to chemotaxis, motility, adhesion, bacterial cell wall properties, secretion, regulating transcription and utilising a substrate in order to establish a successful interaction. Colonisation is mediated by plant receptors and is regulated by the signalling that is connected with phytohormones such as auxin and jasmonic (JA) and salicylic acids (SA). We also highlight changes in the expression of small RNAs and modifications of the cell wall properties. Moreover, in order to exploit the beneficial plant-endophytic bacteria interactions in agriculture successfully, we show that the key aspects that govern successful interactions remain to be defined.

## 1. Introduction

The plant microbiome consists of the total microbial population associated with and interacting with a plant. Part of the microbiome includes the endophytes that colonise the internal parts of plants [1,2]. Although endophytic bacteria primarily occupy the intercellular spaces due to their abundance of the carbohydrates, amino acids and other nutrients, some are also capable of intracellular colonisation. Endophytic strains colonise various parts of plants, including the roots, leaves, stems, flowers and seeds. Nonetheless, plant roots are the organs that are the most abundant in endophytic bacteria, both in terms of their number and diversity. Some endophytic bacteria remain close to their point of entry, whilst others are capable of a systemic spread to other parts of a plant. The latter is especially important as the resulting colonisation of seeds enables their transfer across plant generations. 

Endophytic bacteria are a key determinant of plant health and productivity [3,4,5]. Indeed, seed-borne endophytic bacteria can play a pivotal role in germination, thus enhancing plant survival [6]. The metabolic activity of these microorganisms can result in the mobilisation of nutrients such as PO_4_^3−^ and Fe^3+^, which otherwise would be unavailable to plants. Diazotrophic endophytes, which are capable of nitrogen fixation, are of special interest [7]. Endophytes also have antagonistic effects towards phytopathogens primarily due to their ability to produce chitinases, proteases and siderophores. Alleviating the negative effect of environmental stresses may be the result of the activity of deaminase 1-aminocyclopropane-1-carboxylate (ACC). This can decrease ethylene (ET) production by degrading ACC, which is the precursor of ET that can cause cell necrosis [4]. Numerous studies have shown that endophytic bacteria can increase the resistance of plants to cold and drought and stimulate plant immune system in a process that is called priming [1,8]. Furthermore, plant-related bacteria can degrade the xenobiotics that might otherwise accumulate and reach toxic levels in plant tissues and thereby hinder plant growth [7]. All of the aforementioned properties enable beneficial endophytic bacteria to be utilised not only to increase crop production but to also enhance phytoremediatory efficiencies [9,10]. Unfortunately, efficient host growth promotion is rarely reproducible under field conditions, which exhibit considerable, sometimes undesirable, variation. As a result of such variation, a better understanding of the molecular determinants that underpin plant-endophyte interactions is required [6,11,12].

In this review, we discuss the current state of knowledge regarding the genetic foundations of plant–endophytic bacteria interactions. We highlight the complexity and importance of this interplay based on the latest insights provided by 18 “big-data” based ‘omic approaches and mutational studies where the impact of a discrete component to a particular interaction can be defined. Thus, we considered the increased understanding obtained from the mutants of 52 bacterial strains and 11 plants which have, for example, shed a light on the essential role of phytohormones in regulating endophytic colonisation. To complete the picture, 20 articles undertaking comparative genomic analyses of endophytic bacteria are considered in order to identify conserved and unique genetic determinants of a particular interaction. Our novel synthesis of these diverse datasets provides a more detailed understanding of genetic features involved in endophyte–plant interactions that may pave a way for future development of more successful applications of endophytic bacteria in plant growth enhancement.

## 2. Genetic Features of the Endophytic Bacteria That Are Involved in Interactions with Plants

**Figure 1:**
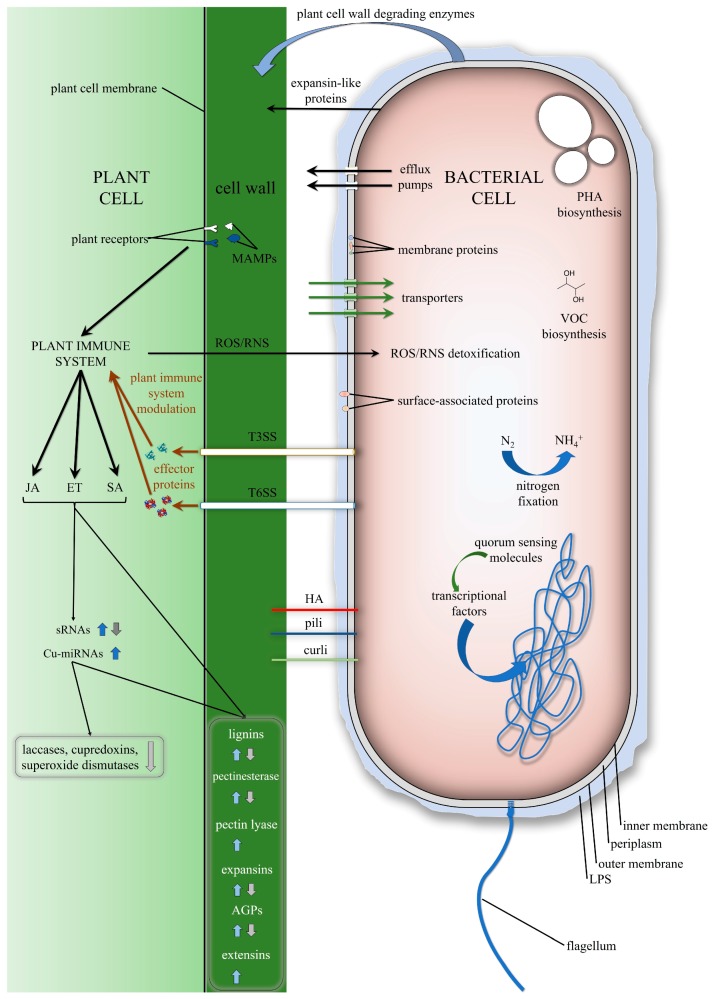
Schematic of plant-endophytic bacteria interactions. Abbreviations used in the figure: polyhydroxyalkanoates (PHA), volatile organic compounds (VOC), reactive oxygen species (ROS), reactive nitrogen species (RNS), type III secretion system (T3SS), type VI secretion system (T6SS), hemagglutinins (HA), small RNAs (sRNAs), copper-micro RNAs (Cu-miRNAs), lipopolysaccharide (LPS), arabinogalactan proteins (AGPs), microbe-associated molecular patterns (MAMPs), jasmonic acid (JA), ethylene (ET), salicylic acid (SA). The arrows pointing upwards indicate an increase, while the ones pointing downwards indicate a decrease in the expression levels.

The colonisation of plants by endophytic bacteria is a complex process that can be divided into five distinct stages: (i) recognising root exudates and motility towards the plant, (ii) adhering to the surface of roots, (iii) biofilm formation, (iv) root surface penetration and (v) colonisation of the internal parts of a plant [4,7]. Each of these stages is mediated by various biomolecules which drive dynamic changes in the expression of the bacterial genes as well as in the colonised plant (Figure 1). In order to determine how these biomolecules exert these effects, it is important that a reductionist approach not be adopted and not to consider individual genes in isolation. Rather, more holistic strategies that employ ‘omic approaches should be considered [13]. Such multicomponent analyses should also be informed by taking into account the key relevant mutants so that any assessments can move beyond finding a simple correlation in order to indicate causative relationships. Furthermore, comparative genomics, which is based on hundreds of sequenced endophytic genomes, enables novel genes that may be putatively important for plant–bacteria interactions to be predicted [14,15]. Studies that have utilised bacterial mutants are presented in Table 1 and transcriptomic, metabolomic and proteomic analyses are presented in Table 2. The major analyses of bacterial comparative genomics are listed in Table 3. Based on these, an overview of the features that are engaged in plant–endophytic bacteria interactions are presented in Figure 1. 

### 2.1. Chemotaxis, Motility

The ability to sense and quickly respond to the signals that are produced by plants is one of the major drivers of successful colonisation by endophytic bacteria. Colonising the roots starts with the chemotaxis of free-living bacteria towards the roots followed by attachment to the rhizoplane. Methyl-accepting chemotaxis proteins (MCPs) play a major role in these first stages. These are transmembrane sensors that allow the molecules surrounding the bacteria to be detected in order to either direct them towards attractants or away from repellents [16]. The involvement of the MCPs in plant colonisation was shown by the *che* inactivation mutant in *Herbaspirillum seropedicae* SmR1. Of the 66 genes encoding for MCPs in SmR1, nine were found to be differentially expressed in the cells that are associated with roots. Inactivation of one of these, Hsero_3720, resulted in a two-fold reduction in the ability of the mutant strains to attach compared to the wild-type strain (Table 1). This MCP is a key transducer that is required to sense rhizosphere and to direct bacteria towards the host-secreted compounds. However, epiphytic and endophytic colonisation by the SmR1 mutant strain remained the same as the wild-type strain [17]. Inactivation of another MCP chemotaxis-like protein that is encoded by *tlp1* gene resulted in the impairment in the taxis to several terminal electron acceptors (oxygen and nitrate) and redox active chemicals as was indicated by a mutant of the rhizospheric strain *Azospirillum brasilense* Sp7. This mutant was deficient in chemotaxis and displayed impaired colonisation of the plant roots [18] (Table 1). 

The movement of bacteria towards the roots is possible because of the presence of flagella and pili. A lack of flagellum synthesis in an *Azoarcus* sp. BH72 mutant impaired its motility although the attachment to the root surface apparently remained unaffected [19]. However, in *A. brasilense* Sp7 the flagellum was essential for adherence to the wheat roots [20]. Defining the exact role for flagella is complicated because its protein constituents are microbe-associated molecular patterns (MAMPs) that elicit resistance. This stated, *Azoarcus* sp. BH72 flagella were not recognised by the plant and failed to elicit a plant defence response [19]. The pili that are produced by *Azoarcus* sp. BH72 also participate in colonising the surface and interior of the roots. The mutational inactivation of the pilus-associated *pilX* gene resulted in a greatly reduced root colonisation due to the impaired twitching motility of the mutant [21]. The significance of the twitching motility was also stressed by generating mutants in other genes coding for parts of the pilus (Table 1). Mutants with no twitching motility had a significant decrease in the colonisation of the surface and interior of the roots [22]. A microscopic observation of the type IV pili of *H. seropedicae* SmR1 during the colonisation of wheat roots further indicated the involvement of this structure in attachment [23]. Some intriguing and superficially contradictory results regarding the involvement of type IV pili were also presented by Cole, et al. [24]. In this study, mutations in the pilus locus increased the colonisation fitness of the rhizospheric strain *Pseudomonas simiae* WCS417r. However, this mutation may promote a planktonic lifestyle, thus resulting in a decrease in cell-to-cell and cell-to-surface interactions and thereby increase motility and colonisation efficiency [24]. 

### 2.2. Adhesion, Biofilm Production

The attachment of bacteria to the root surface is possible, in part, due to the formation of the biofilm, which acts as a physical barrier that protects the embedded bacterial cells. The biofilm that is produced by bacteria comprises water, proteins, polysaccharides, extracellular DNA (eDNA), RNA and ions [25]. In addition to these generic biofilm components, a series of mutants in a range of species has suggested additional contributions from other polymers that affect attachment and colonisation. In *Gluconacetobacter diazotrophicus*, PAL5 exopolysaccharide (EPS) is also involved in forming the biofilm. Inactivation of the *gumD* gene that encodes the enzyme responsible for the first step of EPS biosynthesis resulted in a decrease in the rhizospheric and endophytic colonisation of rice roots by PAL5 [26]. A similar deficiency in the formation of the biofilm and attachment was observed in mutants of the *Rhizobium leguminosarum* biovar *viciae* 3841, which were unable to produce EPS and glucomannan [27] (Table 1). Cellulose is another important component of the biofilm in some species. A *bcsA* gene mutant of *Salmonella enterica* exhibited both a decrease in biofilm formation and, crucially, a reduced ability to colonise its plant host [27,28]. Similarly, inactivation of another gene that is essential for cellulose production (*wssD* gene) in *Herbaspirillum rubrisubalbicans* M1 resulted in a decrease in the attachment to the root surface [29]. In *S. enterica*, a lack of colonic acid (a polysaccharide containing a repeat unit with d-glucose, l-fucose, d-galactose, and d-glucoronate) biosynthesis resulted in a reduced ability to colonise the plants [30]. In *Pseudomonas*, the surface-associated protein LapA is important for biofilm formation and contributes to cell-cell attachment by regulating cell hydrophobicity [31]. In a *lapA* mutant, the initial attachment to the roots was similar to the wild type, but the formation of a microcolony and the subsequent development of a mature biofilm was impaired, which resulted in poorer root colonisation [32] (Table 1). 

Although root exudates can attract a wide variety of bacteria, only those that are able to adhere to the root surface can colonise the plant interior. Adhesion can be mediated by the previously mentioned flagella and pili as well as by specialised proteins such as curli and hemagglutinins [4,7]. Curli proteins affect the adhesion of bacterial cells to various surfaces, cell aggregation and biofilm formation [33]. In *S. enterica*, *agfA* encodes the secreted curli subunit, whilst *afgB* encodes the surface-exposed nucleator around which the curli amyloid fibres form. Inactivation of the *agfB* gene affected the initial attachment as well as the attachment and colonisation over time, whereas inactivation of the *agfA* gene had no such effect [28]. Although hemagglutinins are well known for their role in both plant and human pathogenesis, genes encoding for hemagglutinins are also frequently present in the genomes of endophytic bacteria [34,35,36] and the upregulation of two genes encoding the filamentous hemagglutinin proteins (*Hsero_1294* and *fhaB*) in *H. seropedicae* SmR1 root-attached cells suggest their involvement in attachment to the root surface [23] (Table 2).

### 2.3. Lipopolysaccharide, Membrane Proteins

Components of the bacterial surface play a significant role in the early stages of attachment and colonisation [4]. In Gram-negative bacteria, this is particularly the case for lipopolysaccharide (LPS). LPS consists of three components: (i) lipid A, anchored to the outer bacterial membrane, (ii) a core region and (iii) *O*-antigen, which usually consists of no more than five sugar units [37]. Rhamnose is a monosaccharide that is frequently detected as part of LPS and the *O*-antigen and its biosynthesis requires four genes—*rfbABCD*. Generally, the genes that are involved in LPS biosynthesis are upregulated in early stages of colonisation [21,38]. For example, these genes are found to be upregulated in the presence of maize seedlings as well as flavonoids, naringenin and apigenin, which are commonly found in root exudates. The mutation of one of the genes that is involved in rhamnose biosynthesis (*rfbB* or *rfbC*) resulted in a 100-fold lower level of attachment to the root surface and a lower endophytic colonisation of maize by *H. seropedicae* SmR1 (Table 1). This mutation can also reduce the robustness of the bacterium as was shown by an increased sensitivity towards the detergent sodium dodecyl sulphate (SDS); the antibiotic, polymyxin B sulphate and the phytohormone SA [39]. Similar results in regard to the attachment and colonisation efficiency were observed in the case of the *rfbD* gene mutant of *A. brasilense* [40]. The biosynthesis of *O*-antigen is a complex process and one of its stages is the ligation of the *O*-antigen repeat subunits to the lipid A-core. This reaction is catalysed by *O*-antigen ligase. The gene encoding for this enzyme was upregulated during colonisation of maize roots by *H. seropedicae* SmR1, thereby suggesting its involvement in plant-endophyte interactions (Table 2). Mechanistically, bacterial LPS can bind to the lectin proteins from maize roots, thus resulting in agglutination. Further investigation revealed that a mutant that had been deprived of O-antigen by inactivation of the *O*-antigen ligase failed to interact with the lectin proteins, which resulted in a severe impairment in its attachment to the root surface. Moreover, decreased colonisation efficiencies were observed 10 days after inoculation [41]. 

The membrane proteins of bacteria also play significant roles in plant–endophyte interactions. Amongst these is muropeptide permease, which is necessary for bacterial cell wall peptidoglycan recycling. Inactivation of the gene encoding this permease led to an increased sensitivity to SDS and alterations in the LPS biosynthesis in *H. seropedicae* SmR1. Although the structure of LPS remained the same as in the wild-type strain, it was reduced in quantity and this correlated with a ten-fold reduction in the endophytic colonisation of maize [44]. A proteomic analysis of *G. diazotrophicus* PAL5 in response to plantlets indicated a higher amount of the outer membrane lipoprotein (Omp16) [61] (Table 2). The OprF membrane protein is characteristic for pseudomonads and was also found to aid the attachment to the root surface. This protein is a major porin that facilitates the passage of polar solutes across the outer envelope and participates in maintaining membrane integrity. Inactivation of the *oprF* gene in *Pseudomonas fluorescens* CHA0 resulted in a significant decrease in its attachment to cucumber and tomato roots [45]. 

### 2.4. Plant Cell Wall Modifications

Some bacterial endophytes are capable of producing and secreting plant cell wall-degrading enzymes that are active against cellulose, xylulose and pectins. A local disruption of the plant cell wall can facilitate the entry of bacteria and their spread to other parts of a plant. For example, an *Azoarcus* sp. BH72 mutant devoid of endoglucanase activity had a decreased ability to colonise rice roots and was unable to spread to the aboveground parts of the plant. Moreover, the endoglucanase expression was greatly induced during contact with the rice roots [46] (Table 1 and Table 2). The importance of plant the cell wall-degrading enzymes in the entry of bacteria is further suggested from their upregulation by root exudates. When exposed to root exudates, *Bacillus mycoides* EC18 had an upregulation of the genes encoding hydrolases, pullulanase and a chitin-binding protein (Table 2). *O*-glycosyl hydrolases were also found amongst the upregulated genes, thus suggesting that bacterial cells are capable of utilising plant-derived compounds [62]. 

Although they are rarely found in endophytic genomes, expansins can facilitate cell wall extension (creep) without any actual breakdown or covalent modification of the wall polymers. Thus, an expansin mutant of *Bacillus subtilis* 168 had a significantly reduced ability to colonise roots even though the extension activity in the wild-type strain was very weak when assessed in vitro [48]. All of these observations should be tempered by the fact that the cell wall-degrading enzymes and/or expansins are not required for most successful colonisations because many endophytes enter plants through wounds and natural openings such as the stomata, particularly on the leaves and young stems [63]. Moreover, the genes encoding for the plant cell wall-degrading enzymes are not found in most of the genomes of endophytic bacteria [64,65]. The occurrence of expansins seems to be limited to bacteria from the *Bacillus*, *Xanthomonas*, *Xylella*, *Ralstonia* and *Erwinia* genera [48].

### 2.5. Substrate Utilisation, Transport

Besides being a chemotactic attractant for bacteria, root exudates are also the source of the nutrients that fuel the microbial activities in the rhizosphere and thereby facilitate attachment and internal colonisation. In order to utilise root exudates, bacteria must have adequate transporters and enzymes. Root exudates primarily consist of sugars, polysaccharides, amino acids, aromatic acids, aliphatic acids, fatty acids, sterols, phenolics, plant growth regulators, secondary metabolites, proteins and enzymes [66]. The composition of root exudates changes as plants develop or respond to exogenous stimuli because they are an important part of the plant defence system [67]. One of the compounds that are found in root exudates of maize and lupin is oxalate, which is utilised by bacteria through the activity of oxalate decarboxylase. Inactivation of *oxc*, which encodes oxalate decarboxylase in *Burkholderia phytofirmans* PsJN, reduced the early colonisation of maize and lupin, although the effect was less pronounced in maize (Table 1). This might be explained by the fact that maize roots produce less oxalate than lupin (five-fold less per g of root fresh weight three days after inoculation) [49]. Other nutrients in root exudates include trehalose and maltitol, which are utilised by enzymes encoded by *thuAB* genes to produce their 3-keto derivatives. In *Rhizobium meliloti* 1021, *thuA* and *thuB* genes were induced on the root surface and in the infection threads. Furthermore, disrupting one of these genes led to an impaired root colonisation competence, thus indicating the role of trehalose and/or maltitol utilisation [50,68] (Table 1). Moreover, an analysis of the rhizospheric strain *P. simiae* WCS417r mutants highlighted the importance of the ability to utilise carbohydrate sources in root–bacterial interactions. Mutants that were unable to utilise galactose, galacturonate, glucose, inosine or 2-deoxyribose had reduced colonisation fitness. Interestingly, the results also suggested that auxotrophy for specific amino acids conferred a selective advantage for survival in a plant-associated environment that is abundant in exuded amino acids. This implies that strains can be modified and tailored to respond to specific plant-derived compounds and thereby improve colonisation and promote plant host growth [24]. 

Many bacteria are capable of producing and storing polyhydroxyalkanoates (PHA) such as polyhydroxybutyrate (PHB), which improves survival under stress conditions or in competitive environments. The rhizosphere can be seen as such an environment and carbon storage in the form of PHB can provide a competitive advantage for colonisation. A significant upregulation of the PHB biosynthesis and phasin genes (the latter forming a cover over PHB granules) was observed in the root-attached cells of *H. seropedicae* SmR1. In root-attached bacteria, PHB biosynthesis is stimulated by a low availability of nitrogen and oxygen as well as an excess of carbon sources in root exudates [23]. A high level of the expression of PHA biosynthesis genes was also observed in *A. brasilense* FP2 during the initial steps of wheat-endophyte interactions [38]. In this context, a mutant of *H. seropedicae* SmR1 that was not able to produce PHB was impaired in the early stages of colonisation, which was manifested in a lower number of planktonic and epiphytic cells. Surprisingly, three days after inoculation, no significant differences were observed in the colonisation efficiency between the wild-type strain and the mutant. This observation suggests that PHB may be important until the bacteria start to utilise the plant exudates [17]. Additionally, a lack of PHB production alters the redox balance to increase oxidative stress and also affects the expression of many genes [69]. The importance of PHB was confirmed by field experiments with maize and wheat in which PHA-rich *Azospirillum* cells were more efficient in increasing crop yield [70]. The latter effect was in accordance with the observation that *H. seropedicae* SmR1 mutants that were not able to produce PHB were less capable of promoting plant growth, even though the long-term epiphytic and endophytic colonisation were not affected [71]. 

The plant cell wall can be a source of methanol as a by-product of pectin methylesterase activity. Some endophytic bacteria can utilise methanol as a source of the energy. Mutants of the facultative methylotrophic bacteria *Methylobacterium extorquens* AM1 that were unable to oxidise methanol into formaldehyde or oxidise formaldehyde to CO_2,_ were less able to compete against wild-type cells in colonising the leaves and roots of *Medicago truncatula*. Interestingly, when the mutant and wild-type strains were inoculated separately, each had a wild-type level of plant colonisation. This implies that methanol is an important but not sole source of energy for *M. extorquens* AM1 [51]. This alternative energy source includes ethanol as mutants of *Azoarcus* sp. BH72 that were unable to utilise ethanol exhibited reduction in rice root colonisation. This bacterial property takes advantage of the well-characterised production of ethanol in rice roots under waterlogged and aerated conditions [52]. Also a gene encoding for alcohol dehydrogenase was upregulated in *H. seropedicae* SmR1 cells that were attached to roots [23,72]. In turn, the upregulation of methanol dehydrogenase was observed in *B. mycoides* EC18 exposed to root exudates [62] (Table 2). 

Diazotrophic endophytic bacteria adapt to and modify the plant environment via nitrogen fixation. The enzymes that are involved in nitrogen fixation are encoded by the *nif* genes and transcriptomic analysis has indicated their upregulation when the bacteria attach to the root surface (Table 2). Fixed nitrogen can be supplied to a plant and is one of the most important growth-promoting mechanisms [23,38].

All of the aforementioned nutritive events depend on the substrate transport to allow the bacteria to utilise the plant-derived nutrients in both the rhizosphere and plant interior. This uptake is aided by the upregulation of the genes that are connected with the importers of various compounds as has been observed in many experiments [21,38,62]. For example, the upregulation of membrane porins was observed in the root surface attached cells of *H. seropedicae* SmR1 [17,23].

### 2.6. Stress Protection

During the transition from the host rhizosphere to the endosphere, colonising bacteria must be able to adapt to a new environment that is characterised by a different pH, osmotic pressure and availability of oxygen. They also have to overcome the plant defence responses to an invasion. These include the rapid production of reactive oxygen species (ROS) and reactive nitrogen species (RNS). Thus, endophytic bacteria must detoxify ROS and RNS in order to survive in this challenging environment. ROS- and RNS-scavenging enzymes such as glutathione peroxidase, glutathione S-transferase, catalase and nitric oxide reductase participate in alleviating the harmful effects of stress [7,73]. The importance of ROS-detoxification in the early stages of root colonisation by *G. diazotrophicus* PAL5 was highlighted by superoxide dismutase and glutathione reductase mutants that were not able to colonise roots efficiently [53]. Plant defences can also be withstood via the upregulation of stress-induced protein, stress-response protein and general stress protein as was observed in *B. mycoides* EC18 that had been exposed to root exudates [62]. Plants also produce various phytoalexins that can hinder the growth and survival of rhizospheric and endophytic bacteria. In such cases, one strategy by which bacteria survive is through the activity of efflux pumps. Thus, the SmeDEF efflux pump mutant of *S. maltophilia* D457 had a significant impairment in its ability to colonise the roots. This *smeDEF* efflux pump was upregulated in the presence of root exudates [54]. Another ATP-binding cassette (ABC) multidrug transporter in *H. seropedicae* SmR1 confers a resistance to naringenin, quercetin, JA and SA. Its inactivation results in a significant decrease in roots colonisation [17]. Similar results were observed in the case of the *P. kururiensis* M130 NodT family outer membrane efflux transporter mutant [14]. Furthermore, the upregulation of putrescine ABC transporters was detected in *H. seropedicae* SmR1. This polyamine is involved in the bacterial response to osmotic stress, which can be caused by the higher osmolarity of the rhizosphere compared to the regions that are located away from the roots [23].

Given the necessity to survive the plant defence response, it appears counterintuitive that rhizosphere bacteria can initiate a systemic resistance mechanism. This situation is likely to reflect spatio-temporal differences in the defence responses. This complexity is reflected in the production of siderophores, which are iron chelators and are some of the important plant growth-promoting mechanisms. They are also involved in protecting a plant as they deprive phytopathogens of iron and subsequently stimulate an induced systemic resistance (ISR). The importance of siderophores was demonstrated by the *Serratia marcescens* 90–166 siderophore mutant in which, even though the rhizosphere colonisation remained at the same level as the wild type, that of endophytic colonisation was severely reduced. In such cases, the lack of siderophore production may reduce the ability of bacteria to detoxify active oxygen species, which can use free iron to produce highly toxic hydroxyl radicals via Fenton reactions [56]. Another factor that is important in eliciting a defence and promoting growth is 2,3-butanediol. This bacteria-originating volatile compound elicits the plant defence response against pathogens and also protects bacterial cells against harmful plant root exudates. Thus, a 2,3-butanediol biosynthesis mutant of *B. subtilis* 168 was eliminated from the rhizosphere 21 days after the inoculation, whilst the wild type and 2,3-butanediol overexpressing strains persisted [55].

### 2.7. Bacterial Secretion Systems

The secretion of proteins by bacteria plays a pivotal role in plant-endophyte interactions. Bacteria have different types of secretion systems that are involved in pathogenesis, attachment to the eukaryotic cells, and scavenging resources [86]. Generally, secretion systems primarily enable bacteria to avoid being eliminated by the plant immune system [1]. Eight different types of secretion systems have been described for the Gram-negative (type I secretion system to the type VII secretion system and Sec and Tat) and six (Sec, Tat, SecA2, Sortase, Injectosome and type VIII secretion system) for the Gram-positive bacteria. A type III secretion system (T3SS) can be found in Gram-negative bacteria and delivers effector proteins across the inner bacterial membrane, the periplasmic space and the outer membrane into the cytosol of the eukaryotic cells. The translocated effector proteins can manipulate the host metabolism and the immune system response. *H. rubrisubalbicans* M1 T3SS mutants were less successful in endophytic colonisation [57]. The efficacy of the secreted effector proteins by T3SS is maintained by small cytosolic chaperones [87]. Thus, a mutant of *P. kururiensis* M130 that lacked one of the Tir chaperone proteins had a reduced ability to colonise roots [14]. Interestingly, secretion systems may also delimit endophytic colonisation as was shown by the type VI secretion system (T6SS) mutant of *Azoarcus* sp. BH72, which had a higher colonisation capacity than the wild-type strain. This observation might indicate that some of the effectors that are translocated by T6SS may elicit a local host defence response that limits endophytic colonisation, although this reaction may be host-specific [21]. Transcriptomic analyses further stressed the involvement of secretion systems in host plant-endophytic bacteria interactions (Table 2). *B. phytofirmans* PsJN exhibited the upregulation of the genes of the type II and type IV secretion systems in bacterial cells that were colonising potato [74].

### 2.8. Transcriptional Regulators, Sensor Proteins 

The ability of bacteria to rapidly and precisely respond to environmental changes through transcriptional regulators is essential to migrate towards a plant, attach to it and colonise the host [64]. The regulators of biofilm formation and adhesion are especially vital. These features are often regulated at the level of the bacterial population through quorum sensing. In the well-studied endophyte, *S. maltophilia* R551-3, the quorum sensing molecule DSF (diffusible signal factor) regulates chemotaxis, cell motility, biofilm formation and the multidrug efflux pumps. A mutant that was deficient in DSF production could not form structured cell aggregates and was less efficient in colonising and promoting the growth of its plant host [58]. Similarly, a mutant of *P. fluorescens* 2P24, which was unable to produce another quorum-sensing molecule, acyl-homoserine-lactone, was significantly deficient in colonising the rhizosphere [59]. Similarly, the quorum sensing mutants (*bpI.1* and *bpI.2)* of *B. phytofirmans* PsJN were not able to colonise *Arabidopsis thaliana* [60]. A subsequent study showed the increased expression of *bpI.2* in *B. phytofirmans* PsJN when colonising potato plants [74]. 

Other more general transcriptional regulators are implicated in plant–endophytic bacteria interactions. The mutation of *rpoS*, which is a general stress response regulator sigma factor, led to a decrease in the attachment to the plant roots in *S. enterica*. Although RpoS is known to regulate the genes that are engaged in forming the biofilm and producing adhesins, the *rpoS* mutation had minimal effect on the biofilm formation. Analysis of a *rpoS* mutant strain indicated a lack of curli and cellulose production but complementation with *rpoS* failed to restore their production, even though attachment to the roots was observed. Such observations suggest that some other adhesins are important for the initial attachment in *S. enterica* [28]. An increased expression of another sigma-28 factor was observed in the endophytic strain *B. mycoides* EC18 when responding to root exudates. The sigma-28 factor is involved in regulating the flagellin, chemotaxis and motility-related genes [62]. Another important transcription factor in plant-bacteria interactions is GreA, which was observed in the proteome of the *G. diazotrophicus* PAL5. Its importance was also shown in a GreA mutant of *Rhizobium* that was not able to establish effective symbiosis possibly due to an alteration in its ability to adapt to hyperosmotic and salt stresses [61].

The cyclic di-GMP (c-di-GMP) is a secondary messenger in bacteria that regulates various behaviours, among which it is the key driver of lifestyle switch between the motile free cells and formation of the biofilm [88]. Diguanylate cyclase/phosphodiesterase with a PAS/PAC sensor is a bi-functional enzyme that is involved in forming and degrading c-di-GMP. Its deletion in the genome of the *Azoarcus* sp. BH72 resulted in reduced root colonisation, possibly due to an alteration in the c-di-GMP levels. Similar results were observed for a mutant with an inactivated GGDEF domain-containing protein [21]. 

Comparative genomics of bacterial genomes have suggested that endophytic behaviour is connected with the multiplication of the genes encoding transcriptional regulators [7,64] (Table 3). Analysis of bacterial genomes revealed an enrichment of the transcription regulators from the LacI-family in plant-associated bacteria. This was coupled with an enrichment of the LacI-family controlled regulons, which are primarily involved in carbohydrate metabolism and transport [14]. Moreover, a comparative transcriptomic study of the endophytic strain *B. mycoides* EC18 and soil isolates revealed differences in the expression of transcriptional regulators in response to root exudates. Five transcriptional regulators were upregulated in the endophytic strain, including IclR, which is related to multidrug resistance and the degradation of aromatic compounds as well as sigma-28 factor, which is involved in regulating the flagellin gene, chemotaxis and motility [62]. There was also a downregulation of some of the transcriptional factors that may be beneficial for plant-endophyte interactions. For example, the downregulation of the two-component transcriptional regulator QseB1 in response to root exudates was observed for *Azoarcus* sp. BH72. This protein is involved in the transcriptional regulation of the flagella regulon, which was also downregulated in response to root exudates. While flagella-based movements and attachment to the surface is important in the initial colonisation, downregulation can have positive results for bacteria as the flagellum can also be recognised by a plant as a MAMP in order to elicit a defence response [21].

The results of other experiments have indicated the upregulation of various sensors such as NarX/NarL, which is a classic two-component system that is based on a membrane sensor protein (NarX) and DNA-binding regulator (NarL), in response to a plant presence. This sensor system regulates the respiratory membrane-bound nitrate reductases [38]. An increased expression of the genes encoding Fnr-like proteins was observed in the *H. seropedicae* SmR1 in which the *fnr1* and *fnr2* genes acted as intracellular redox sensors and regulated the expression of other genes according to changes in oxygen levels [23].

## 3. Plant Genetic Features that are Involved in Interactions with Endophytic Bacteria

Endophytic interactions are mediated through various receptors and changes in the plant hormone signalling pathways. Some changes in the expression profiles are also mediated through small RNAs. The initial stages of colonisation by bacteria result in various changes in the plant cell wall and lignification of the cell wall. The relevant ‘omic studies are summarised in Table 2 whilst those utilising plant mutants are listed in Table 4.

### 3.1. Plant Receptors

The ability of plants to recognise bacterial signals is primarily mediated by the family of receptor-like kinases (RLK), which include a leucine-rich repeat that contains receptor-like kinases (LRR-RLKs), wall-associated kinases (WAK), lectin receptor-like kinases (LecRLKs) and Lys-motif receptors (LysM). These receptors are well known for their involvement in recognising phytopathogens but only a few studies have pointed out their relevance in identifying beneficial endophytes [102]. For example, the upregulation of two genes encoding for NBS-LRR proteins were found in *T. aestivum* that had been inoculated with *A. brasilense* FP2 [38]. An analysis of the response of sugarcane to the endophytic diazotrophic bacteria revealed the downregulation of a SHR5 receptor that belongs to the LRR-RLK family [103]. Another LRR-RLK receptor, FLAGELLIN SENSING 2 (FLS2), is involved in recognising MAMP and binds to flg22, which is a 22-amino-acid peptide that is present in the N-terminal part of a flagellin. Recognising flg22 leads to rapid extracellular alkalisation, ROS production, the activation of a mitogen-activated protein kinase (MAPK) cascade and the upregulation of pathogenesis-related genes in *A. thaliana* [104,105]. flg22 from the rhizobacterium *B. phytofirmans* had weak elicitory activity on grapevine but a potent activity on *A. thaliana*. Further investigation revealed that the FLS2 receptor from grapevine had evolved in order to specifically distinguish the flagellin that had originated from the grapevine-associated *B. phytofirmans* [104].

### 3.2. Plant Hormone-Signalling Pathways

Phytohormones and their related signalling pathways play a pivotal roles in plant defence, and therefore, it is unsurprising that they are also involved in interactions with endophytic bacteria [106]. Studies have indicated a role for ET, SA and JA in regulating endophytic colonisation and the diversity of endophytic bacterial populations. For example, an endophytic colonisation of *M. truncatula* by *K. pneumoniae* 342 led to the activation of the ET signalling pathway and an ET-insensitive mutant of *M. truncatula* was hyper-colonised by the endophytic strain [107]. In agreement with these results, sugarcane that had been colonised by diazotrophic endophytes, *G. diazotrophicus* PAL5 and *H. rubrisubalbicans* HCC103, exhibited an increased expression of a putative ET receptor (SCER1) at 24 h as well as seven days after the inoculation. Conversely, a decrease in the expression of SCER1 was observed after inoculation with pathogenic bacteria and virus [108]. It could be that an increase in the ET receptors reduces the host’s sensitivity to ET in order to lower the plant defence mechanisms and permit colonisation [108]. Notably, in *Nicotiana attenuata* mutants that were impaired in ET biosynthesis (*ir-aco1*) or perception (*35S-etr1*), the diversity of the culturable community of the root bacteria was lower than in the wild-type plants [109]. 

Another study showed that a mutant of *A. thaliana* with a disrupted SA-mediated and SA-independent defence response was hypercolonised by *K. pneumoniae* 342 and *S. enterica* serovar Typhimurium 14,028 [107]. Higher internal colonisation by *G. diazotrophicus* PAL5 and *S. enterica* serovar Typhimurium 14,028 was also found in an *A. thaliana* mutant that was defective in the SA-mediated defence. Surprisingly, this deficiency had no effect on the colonisation level of *K. pneumoniae* 342 [107,110]. As with other defence hormones, the disruption of the SA-mediated and SA-independent defence responses reduces the diversity of an endophytic bacterial community [111]. Similarly, *A. thaliana* plants with an altered SA signalling were found in different root microbiomes compared to the wild type in terms of the relative abundance of specific bacterial families [106]. Work on *P. putida* BP25 that was colonising *A. thaliana* showed that the bacteria trigger SA signalling, which in turn regulates colonisation [112].

In the case of *Azoarcus* sp. BH72 colonised rice roots, the induction of JA signalling was observed [85]. JA biosynthesis mutants (*fad3/7/8*) showed more epiphytic colonisation. However, the activation of the JA signalling pathways reduced bacterial diversity of the root endophytes of wheat, while the microbiome in the rhizosphere and shoot endosphere were unaffected [114]. Contrasting results were shown by Carvalhais, et al. [115] who worked on the rhizosphere bacterial communities of *A. thaliana* in which the activation of the JA defence pathways altered the bacterial composition of the rhizosphere. Moreover, an enrichment of the bacterial species that are closely related to one that is known to be involved in plant defence was also observed. This suggested the intriguing possibility that plants may recruit microbes on an as-needed basis [115]. This was supported by studies undertaken by Kwak, et al. [116], who revealed that tomato cultivars were capable of recruiting specific flavobacteria from the soil, which made them resistant to the soil-borne pathogen *Ralstonia solanacearum*. 

### 3.3. Small RNAs

Small RNAs (sRNAs) and among them micro RNAs (miRNAs) are known to be important post-transcriptional regulators of gene expression. The sRNAs affect plant growth and development, their responses to abiotic stresses and phytopathogens [102]. Recent studies also indicated their involvement in interactions with endophytic bacteria. For example, the inoculation of *T. aestivum* with the endophytic rhizobium *Azorhizobium caulinodans* ORS571 resulted in an altered miRNA expression. The peak response was found at 12–24 h after inoculation and the responses in the roots and shoots differed. The roots seemed to be more sensitive to the inoculation than the shoots, possibly because this strain colonises roots [117]. Another study by Thiebaut, et al. [118] focused on the response of maize sRNAs to inoculation with the diazotrophic bacteria *H. seropedicae* and *A. brasilense* and showed the upregulation of the copper-miRNAs (Cu-miRNAs): miR397, miR398, miR408 and miR528 coupled with an inhibition of their targets. These miRNAs are called Cu-miRNAs because they target genes that encode proteins with a Cu cofactor such as laccases, superoxide dismutases and cupredoxins. These enzymes participate in generating an oxidative burst and response signalling, both of which are rapid responses to a pathogen challenge [118,119]. Their downregulation suggests that both diazotrophic strains suppress the early plant defence response [118].

### 3.4. Cell Growth, Expansion 

Transcriptional analyses of various plant species that had been inoculated with endophytic bacteria have indicated the upregulation or downregulation of the genes that are related to cell wall modifications, which are likely to aid in the colonisation process. These include hydroxyproline-rich glycoproteins (HRGPs), expansins and pectinesterases. HRGPs have been implicated in many biological functions and are usually divided into three complex multigene families, i.e., (i) arabinogalactan proteins (AGPs), (ii) extensins and (iii) proline-rich proteins [120]. The involvement of extensins in interactions with pathogenic and beneficial bacteria is well characterised. Following challenge with pathogens, increased expression of extensins plays an important role in plant defence by strengthening the cell wall. Interestingly, increases in extensins were also found in nodules that had been colonised by symbiotic *R. leguminosarum*. Extensins form part of the root mucilage along with AGPs, pectic polysaccharides, secondary metabolites, antimicrobial compounds and extracellular DNA, and play a key role in root defence through the formation of a root extracellular trap. This structure can simultaneously enclose phytopathogens and attract beneficial microbes [121]. AGPs also influence the rhizosphere microbiome; for example, by enriching the rhizospheric bacteria that can hydrolyse and metabolise AGPs-derived sugars for their growth [122]. Developmentally, expansins facilitate the loosening of the cell wall components during division and pectinesterases catalyse the de-esterification of polygalacturonans, which could also be features of endophytic interactions [80]. Thus, upregulation of the expansin gene expression was observed in rice roots following inoculation with bacteria from the *Azospirillum* species. Interestingly, the identical upregulation of this expansin was observed regardless of the type of colonisation—rhizospheric or endophytic [77]. An increased expression of expansins was also observed in *N. tabacum* that had been colonised by the rhizospheric strain *B. subtilis* OKB105 or in wheat roots in response to an internal colonisation by *A. brasilense* FP2 [123]. Such changes could be linked to the downregulation of the genes encoding for fasciclin-like AGP and pectinacetylesterase [38]. Intriguing results regarding expansins were obtained from a transcriptomic analysis of cotton roots in response to an endophytic colonisation in which the expression of two expansins was upregulated while another two were downregulated. The gene encoding pectinesterases, pectate lyase, fasciclin-like AGPs, extensin, cellulose synthase and COBRA-like proteins was among the upregulated genes [80]. The latter could be of particular importance as COBRA-like proteins have been proven to be key regulators in the orientation of cell expansion and the status of cellulose crystallinity [124]. In addition, the upregulation of the cell wall loosening enzymes may facilitate endophytic penetration and systemic endophytic colonisation. This may result in an enhanced growth of the roots and thus represents a mechanism for promoting plant growth. It also seems that these changes in the cell wall enzymes are not universal as no changes were observed in the transcriptome of some plants [78,79]. 

### 3.5. Lignin Biosynthesis

Lignin is one of the main components of the plant cell wall and plays a pivotal role in growth, development, lodging resistance and responses to biotic and abiotic stresses. The accumulation of lignin in the cell wall is an important response to phytopathogens and is a barrier against the spread of bacterial cells. It also reduces the infiltration of fungal enzymes and toxins into the plant cell walls [125]. Given this, it is perhaps surprising that endophytic colonisation can result in an accumulation of lignin. For example, a higher lignin content was observed in the roots of cotton after inoculation with *B. amyloliquefaciens* pb1 [80]. An increase in the expression of the cell wall bound peroxidase that is involved in the lignification of the cell walls was found in *A. thaliana* in response to an endophytic colonisation by *P. putida* BP25 [112]. An increased expression of cinnamyl alcohol dehydrogenase (CAD), which is involved in lignin biosynthesis, was found in *M. sinensis* as a response to an endophytic colonisation by *H. frisingense* GSF30^T^ [79]. It may be that the pattern and/or type of lignin that is produced in response to endophytes are distinctive. On the other hand, the expression of CAD was downregulated in wheat that had been inoculated with *A. brasilense* FP2 [38]. An analysis of the miRNAs expression profile of maize in response to diazotrophic bacteria indicates reduced lignin biosynthesis because the induction of miR408 was followed by the downregulation of its targets—laccases [118]. Therefore, miRNA expression seems likely to be the means by which these endophytes manipulate lignification in the host. 

## 4. Conclusions

The purpose of this review was to summarise the key aspects of plant–endophytic bacteria interactions as revealed by bacteria and plant mutants, comparative genomics and other ‘omics approaches. We highlight the fact that the interplays between plants and endophytic bacteria are complex and are still far from being fully elucidated. In particular, the mechanisms through which endophytic bacteria avoid or neutralise their plant host needs to be further explored. Although some of the mechanisms of plant–endophytic bacteria interactions are known, it is also essential to consider their temporal and spatial dimensions. As most of the endophytes are recruited from the soil, plant exudates act as a chemoattractant luring bacteria capable of exudate detection and facilitating their movement towards the roots. Root exudates induce wide array of changes in the bacteria transcriptome, such as upregulation of genes encoding efflux pumps, transporters and enzymes. This are accompanied by the changes in the bacterial cell wall properties. Adhesion to the rhizoplane, mediated by the pili, hemagglutinins and curli is followed by biofilm formation, which allows establishment of a permanent colony. Internal colonisation that may be feasible due to the cell wall-modifying enzymes, induces a response of the plant immune system. The specific receptors recognise MAMPs, like LPS and flagella, eliciting plant immune responses, mainly ROS generation. That in turn, enforces expression of detoxication proteins in bacteria. Simultaneously, endophytes are capable of plant immune system mediation through effector proteins translocated by the secretion system. The successful colonisation is regulated by phytohormones, for example ET, JA and SA, which influence expression of sRNA and numerous genes. Observed changes in the plant cell wall may facilitate further colonisation and spreading to the upper parts of the plant.

A greater understanding of plant–endophytic bacteria interaction is vital for the successful inoculation of plants with growth-promoting endophytes, which can result in a tangible increase in crop health and yield. Various analyses have indicated that there is no single gene or set of genes that is responsible for the endophytic behaviour of bacteria. As such, ‘omics analyses coupled with comparative genomics of bacterial strains are indispensable in the attempts to untangle the underpinning molecular determinant of endophyte–plant interactions. However, ‘omics analyses to date usually focus on single time points and on endophyte or plant host response, failing to capture dynamic interplays. This would involve a novel, dual transcriptomic approach where gene expression profiles of the endophytic strain and plant host would be described. If combined with a focus on model plants such as *A. thaliana* and *Brachypodium distachyon,* this would facilitate the temporal and spatial dimensions of these interactions being described. To move beyond correlation, further studies should exploit the genome editing of these model plants in order to clearly define the function of the key genes that are targeted through such complex analyses. With the now well-established techniques of site-directed mutagenesis using such as CRISPR/Cas9, this objective is entirely feasible. Comparative genomics studies including newly sequenced bacterial endophyte genomes should focus on prediction of genes putatively involved in interaction with the host. A good example of this an approach was provided by Levy, et al. [14] involving 3837 bacterial genomes that target numerous genes whose relevance for interactions with the host was confirmed by inactivation mutants. Screening collections of endophyte inactivation mutants could also enable identification of mutants showing higher colonisation capabilities, as shown for some auxotrophic strains. This opens fascinating opportunities for genetic manipulation of strains with greater plant growth-promoting abilities which could be efficiently used under field conditions. While in recent years great progress has been made in this area, further research is needed, especially on the environmental impact of plant interactions with endophytes. Expanding this knowledge may even result in at least partial substitution of fertilisers and pesticides with more eco-friendly agricultural practices that involve the use of endophytic bacteria.

## Figures and Tables

**Table 1:** The bacterial mutants that were used in studies dedicated to understanding the genetic foundations of beneficial plant–bacteria interactions.

Bacterial Strain(s)	Gene (Accession Number)	Encoded Feature/Characteristic	Plant(s)	Major Findings	References
Chemotaxis, Motility					
*H. seropedicae* SmR1	(Hsero_3720)	methyl-accepting chemotaxis transducer transmembrane protein	*Zea mays*	decreased chemotaxis towards the plant and attachment to the roots	[17]
*P. fluorescens* OE28.3, SBW25, F113 and WCS365	*cheA*	chemotaxis protein	*Solanum lycopersicum*	ten- to 1000-fold decrease in the ability to colonise the root tip	[42]
*A. brasilense* Sp7	*tlp1*	transducer-like protein 1, chemoreceptor-like protein	*Triticum aestivum*	significant reduction in root colonisation	[18]
*A. brasilense* Sp7	*mot3*	bacterial flagellar motility	*T. aestivum*	significant decrease in adsorption capacity to roots	[20]
*Azoarcus* sp. BH72	*fliC3* (azo2704)	major structural protein flagellin	*Oryza sativa* subsp. *indica* cv. IR36	significant reduction in root colonisation	[19]
*Azoarcus* sp. BH72	*pilX* (azo2916)	type IV fimbrial biogenesis protein PilX	*O. sativa* ssp. *japonica* cv. Nipponbare	reduced root colonisation	[21]
*Azoarcus* sp. BH72	*pilA* (azo3355)	major structural component of the pilus structure	*O. sativa* subsp. *indica* cv. IR36	strong reduction in endophytic and surface colonisation	[22]
*Azoarcus* sp. BH72	*pilT* (azo3468)	type IV pilus retraction protein	*O. sativa* subsp. *indica* cv. IR36	strong reduction in endophytic colonisation, 50% reduction in surface colonisation	[22]
Adhesion, biofilm formation					
*Pseudomonas putida* KT2440	*lapA*	surface adhesion protein	*Z. mays*	impaired root colonisation	[32]
*Salmonella enterica* serovar Enteritidis	*agpB*	curli subunit	*Medicago sativa*	reduced attachment capacity to the roots	[28]
*G. diazotrophicus* PAL5	*gumD* (YP_001602791)	polysaccharide biosynthesis glycosyltransferase, exopolysaccharide biosynthesis	*O. sativa*	defective rice root surface attachment, reduced root surface and endophytic colonisation	[26]
*R. leguminosarum* biovar *viciae* 3841	*gmsA* (CAK07156.1)	glucomannan production protein	*Pisum sativum*	defective attachment and biofilm formation on the root hairs	[27]
*R. leguminosarum* biovar *viciae* 3841	*pssA* (CAK09242.1)	CDP-diacylglycerol-serine O-phosphatidyltransferase, acidic exopolysaccharide biosynthesis	*P. sativum*	defective attachment to the root hairs	[27]
*S. enterica*	*yihO*	glucuronide transporter, O-antigen capsule assembly and translocation, colonic acid biosynthesis	*M. sativa*	reduced ability to attach and colonise sprouts	[30]
*R. leguminosarum* biovar *viciae* 3841	*celA* (CAK07141.1); *bcsA* according to the nomenclature by Romling and Galperin [43]	cellulose synthase catalytic subunit [UDP-forming], cellulose biosynthesis	*P. sativum*	deficient biofilm formation on the roots	[27]
*S. enterica*	*bcsA*	cellulose synthase catalytic subunit [UDP-forming], bacterial cellulose synthesis	*M. sativa*	reduced ability to attach and colonise sprouts	[30]
*H. rubrisubalbicans* M1	*wssD* (Hrubri_1119); *bcsZ* according to the nomenclature by Romling and Galperin [43]	beta-1,4-glucanase (cellulase) (EC 3.2.1.4), part of the cellulose biosynthesis operon	*Z. mays*	decrease root surface attachment and endophytic colonisation	[29]
Lipopolysaccharide, membrane proteins					
*H. seropedicae* SmR1	*rfbB* (Hsero_4410)	dTDP-D-glucose 4,6-dehydratase, biosynthesis of rhamnose	*Z. mays*	100-fold lower attachment to the root surface, decreased efficiency in endophytic colonisation	[39]
*H. seropedicae* SmR1	*rfbC* (Hsero_4411)	dTDP-4-keto-6-deoxy-D-glucose 3,5-epimeras, biosynthesis of rhamnose	*Z. mays*	100-fold lower attachment to the root surface, decreased efficiency in endophytic colonisation	[39]
*A. brasilense*	*rfbD* (AMK58_RS28935)	dTDP-4-dehydrorhamnose reductase, biosynthesis of rhamnose	*Z. mays cv.* ‘Funk’s Tronador G422T’	impaired attachment to the roots and decreased root colonisation	[40]
*H. seropedicae* SmR1	*waaL* (Hsero_3570)	O-antigen ligase, catalyse a key step for LPS biosynthesis	*Z. mays*	severely impaired in colonisation	[41]
*H. seropedicae* SmR1	*ampG* (YP_003773531.1)	muropeptide permease of the major facilitator superfamily, recycling of the cell wall peptidoglycan	*Z. mays*	10-fold decrease in endophytic population in roots one and three days after inoculation	[44]
*P. fluorescens* CHA0	*oprF* (EF592174)	outer membrane porin F	*Cucumis sativus* var. Poinset 76 and *S. lycopersicum* Platense	decrease in adsorption capacity to roots	[45]
Plant cell wall modifications					
*Azoarcus* sp. BH72	*eglA* (azo2236)	beta-1,4-glucanase (cellulase) (EC 3.2.1.4)	*O. sativa* subsp. *indica* cv. IR36	decrease of endophytic colonisation	[46]
*Bacillus amyloliquefaciens* TB2	*eglS*	endo-beta-1,4-glucanase	*Brassica rapa* subsp. *pekinensis* and *chinensis*	decrease of endophytic colonisation	[47]
*B. subtilis* 168	*yoaJ*	expansin, causes loosening and extension of plant cell walls by disrupting the non-covalent bonding between the cellulose microfibrils and matrix glucans	*Z. mays*	significant decrease in ability to colonise roots	[48]
Substrate utilisation, transport					
*B. phytofirmans* PsJN	*oxc*	oxalate decarboxylase	*Lupinus albus* L., cv. *Amiga, Z. mays* subsp. *mays*, cv. Birko	impaired early colonisation	[49]
*R. meliloti* 1021	*thuA*	trehalose utilisation-related protein	*M. sativa*	impaired colonisation of roots	[50]
*R. meliloti* 1021	*thuB*	trehalose utilisation-related protein	*M. sativa*	impaired colonisation of roots	[50]
*H. seropedicae* SmR1	*phbC1* (Hsero_2999)	polyhydroxybutyrate (PHB) synthase subunit C	*Z. mays*	eight-fold fewer planktonic and epiphytic cells during the early stages of root colonisation	[17]
*M. extorquens* AM1	*mxaF* (MexAM1_META1p4538)	methanol dehydrogenase subunit alpha, oxidise methanol to formaldehyde (together with MxaI)	*M. truncatula*	decreased colonisation abilities and persistence in different parts of the plant	[51]
*M. extorquens* AM1	*mptG* (MexAM1_META1p1760)	beta-ribofuranosyl aminobenzene 5’-phosphate synthase, involved in tetrahydromethanopterin biosynthesis	*M. truncatula*	decreased colonisation abilities and persistence in different parts of the plant, increased sensitivity to formaldehyde and methanol	[51]
*Azoarcus* sp. BH72	*exaA2* (azo2972)	quino(hemo)protein alcohol dehydrogenase, pyrroloquinoline quinone (PQQ)-dependent (EC 1.1.2.8)	*O. sativa* subsp. *indica* cv. IR36	decrease in root colonisation	[52]
*Azoarcus* sp. BH72	*exaA3* (azo2975)	quino(hemo)protein alcohol dehydrogenase, PQQ-dependent (EC 1.1.2.8)	*O. sativa* subsp. *indica* cv. IR36	decrease in root colonisation	[52]
Stress protection					
*G. diazotrophicus* PAL5	*sodB* (GDI_2168)	superoxide dismutase [Mn/Fe] (EC 1.15.1.1)	*O. sativa* subsp. *indica* cv. IR42	decrease in number of tightly root-associated bacterial and endophytic colonisation	[53]
*G. diazotrophicus* PAL5	*gor* (GDI_2216)	glutathione reductase (EC 1.8.1.7)	*O. sativa* subsp. *indica* cv. IR42	decrease in number of tightly root-associated bacterial and endophytic colonisation	[53]
*Stenotrophomonas maltophilia* D457	*smeE* (SMD_3657)	RND efflux system, inner membrane transporter, part of the SmeDEF efflux pump involved in quinolone resistance	*Brassica napus* cv. Californium	impaired colonisation of roots	[54]
*H. seropedicae* SmR1	(Hsero_4782)	ABC-type multidrug transporter	*Z. mays*	20-fold lower endophytic and epiphytic populations three days after inoculation	[17]
*Paraburkholderia kururiensis* M130	(2553408008)	outer membrane efflux transporter, nodT family	*O. sativa* var. BALDO	four- to six-fold decrease ability to colonise roots	[14]
*B. subtilis* 168	*pta, als*	2,3-butanediol biosynthesis	*Capsicum annuum*	decreased ability to persist in the rhizosphere	[55]
*S. marcescens* 90-166	*entA* (AAY84_01970)	2,3-dihydro-2,3-dihydroxybenzoate dehydrogenase, enterobactin biosynthesis	*Cucumis sativus*	significant decrease in root population	[56]
Bacterial secretion systems					
*H. rubrisubalbicans* M1	*hrcN* (Hrubri_2444)	T3SS ATPase	*O. sativa*	100-fold decrease in endophytic population in roots nine days after inoculation	[57]
*H. rubrisubalbicans* M1	*hrpE* (Hrubri_2433)	T3SS structural apparatus	*O. sativa*	100-fold decrease in endophytic population in roots nine days after inoculation	[57]
*Azoarcus* sp. BH72	*ppkA* (azo3888)	T6SS serine/threonine protein kinase	*O. sativa* ssp. *japonica* cv. Nipponbare	increased colonisation capacity	[21]
*P. kururiensis* M130	*cesT* (2553406074)	Tir chaperone protein	*O. sativa* var. BALDO	four-six-fold decrease in the ability to colonise roots	[14]
Transcriptional regulators, sensor proteins					
*S. maltophilia* R551-3	*rpfF* (Smal_1830)	enoyl-CoA hydratase, synthesis of quorum sensing molecule - diffusible signal factor (DSF)	*B. napus* cv. Californium	decreased colonisation efficiency and plant growth promotion	[58]
*P. fluorescens* 2P24	*pcoI*	acyl-homoserine-lactone synthase, quorum sensing molecules biosynthesis	*T. aestivum*	deficiency in colonisation of rhizosphere	[59]
*B. phytofirmans* PsJN	*bpI.1* (Bphyt_0126)	AHL synthase of chromosome 1 QS system	*A. thaliana* Col-0	decreased root colonisation	[60]
*B. phytofirmans* PsJN	*bpI.2* (Bphyt_4275)	AHL synthase of chromosome 2 QS system	*A. thaliana* Col-0	decreased root colonisation	[60]
*Azoarcus* sp. BH72	(azo1544)	GGDEF/EAL/PAC/PAS-domain-containing protein	*O. sativa* ssp. *japonica* cv. Nipponbare	reduced root colonisation	[21]
*Azoarcus* sp. BH72	(azo2408)	GGDEF domain-containing protein	*O. sativa* ssp. *japonica* cv. Nipponbare	decreased root colonisation	[21]
*S. enterica* serovar Newport	*rpoS*	stationary-phase sigma factor, regulating biofilm formation, *agfD* and other adhesins	*M. sativa*	decreased colonisation of sprouts	[28]

**Table 2:** The transcriptomic, metabolomic and proteomic studies on plant-endophytic bacteria interactions.

Type of Analysis	Strain(s)	Plant(s)	Major Findings	References
Transcriptomic analysis of *Azoarcus* sp. BH72 in response to the root exudates	*Azoarcus* sp. BH72	*O. sativa* cv. Nipponbare	changes in the expression of 176 genes, primarily those encoding for hypothetical proteins, which are the proteins that are involved in the metabolic processes, especially producing and conserving energy, amino acid transport and metabolism	[21]
Transcriptomic analysis of *H. seropedicae* SmR1 of bacteria attached to the roots of *T. aestivum* cv. CD104	*H. seropedicae* SmR1	*T. aestivum* cv. CD104	upregulation of the genes related to the type IV pili, PHB biosynthesis, nitrogen fixation, stress tolerance, adhesion and utilisation of the plant-derived compounds	[23]
Transcriptomic analysis of *B. mycoides* EC18 and *B. mycoides* SB8 in response to the root exudates	*B. mycoides* EC18 (endophyte) and *B. mycoides* SB8 (soil bacteria)	*Solanum tuberosum* cv. Seresta	more pronounced response of the endophytic strain to root exudates, upregulation of the genes related to amino acid metabolism, membrane proteins, transcriptional regulators, stress-related genes and plant cell wall-degrading enzymes in the endophytic strain	[62]
Transcriptomic analysis of *B. phytofirmans* PsJN colonising potato plant	*B. phytofirmans* PsJN	*S. tuberosum* cv. Bionta	expression of the genes related to regulating transcription, general metabolism (sugars, amino acids, lipids and nucleotides), secretion systems, energy production and cellular homeostasis	[74]
Transcriptomic analysis of *H. seropedicae* SmR1 in response to the naringenin	*H. seropedicae* SmR1	-	changes in the expression profile of the genes related to the bacterial cell wall, repression of the genes related to the chemotaxis, flagella biosynthesis, downregulation of the genes related to amino acid and sugar transport, upregulation of the multidrug transport efflux encoding genes	[75]
Transcriptomic analysis of *P. kururiensis* M130 in response to the plant extract	*P. kururiensis* M130	*O. sativa* var. BALDO	diversified expression of the genes related to the cellular processes, metabolism, secretion and transport; diversified expression of potential antisense RNAs (asRNAs) and sense mRNA that can possibly be affected by asRNAs	[76]
Transcriptomic analysis of *A. brasilense* FP2 and *T. aestivum* (dual RNA sequencing)	*A. brasilense* FP2	*T. aestivum* cv. CD-104	bacterial response: high expression of the surface layer protein (*sbpA*), ABC sugar transporters (*gguA* and *gguB*), monosaccharide transporters, calcium-binding proteins, superoxide dismutase and proteins related to polysaccharides, LPS biosynthesis, transport of exopolysaccharides, PHAs biosynthesis and nitrogen fixation; plant response: differential expression of 776 of the expressed sequence tag that are involved in transport, biological regulation, defence mechanisms, production of phytohormones and phytochemicals	[38]
Transcriptomic analysis of *O. sativa* roots in response to inoculation with bacteria	*A. lipoferum* 4B (isolated from Cigalon), *Azospirillum* sp. B510 (isolated from Nipponbare)	*O. sativa japonica* cv. Cigalon and Nipponbare	diversified expression of 7384 genes of rice after inoculation with bacteria, primarily those involved in primary metabolism, transport, regulating transcription and protein fate, 34 genes similarly regulated by both strains, among them the pathogenesis-related gene (PR-10); B510 strain leads to the repression of a larger number of the genes that are related to stress and plant defence than the 4B strain	[77]
Transcriptomic analysis of *Saccharum officinarum* inoculated with bacteria and uninoculated specimens that were subjected to water depletion for three days	*G. diazotrophicus* PAL5	*S. officinarum* cv. SP70-1143	higher level of colonisation by the endophytic strain of water-deficit roots; higher tolerance to drought stress of the inoculated plants; diversified expression of drought-related genes in the inoculated plants	[78]
Transcriptomic and proteomic analyses of *Miscanthus sinensis* three hours and three weeks after inoculation	*H. frisingense* GSF30^T^	*M. sinensis*	prominent upregulation of the genes involved in jasmonate signalling and biosynthesis in the early (3 h) response to the inoculation; upregulation of the ethylene receptor and downregulation of the ethylene response factor after three weeks	[79]
Transcriptomic analysis of cotton roots ten days after inoculation with bacteria	*B. amyloliquefaciens pb1*	*Gossypium* sp.	upregulation of the genes related to nitrate assimilation, cell growth, transport, hormones, transcription factors and antioxidants	[80]
Metabolomic analysis of *Z. mays* roots and leaves in response to inoculation with bacteria	*H. seropedicae* SmR1 and SmR54, *A. brasilense* FP2 and FP10	*Z. mays* FV252 and FV2	maize genotype-specific pattern of accumulation of metabolites, primarily changes in the mannitol, trehalose and isocitrate concentrations	[81]
Metabolomic analysis of poplar in response to *Paenibacillus* sp. P22	*Paenibacillus* sp. P22	*Populus alba* × (*P. davidiana* + *P. simonii*) × *P. tomentosa*]	increased concentration of asparagine, urea and threitol; depletion of sugars and organic acids of the tricarboxylic acid cycle in the plants that had been inoculated with bacteria	[82]
Metabolomic analysis of *Vitis vinifera* roots and stems in response to *Enterobacter ludwigii* EnVs6	*E. ludwigii* EnVs6	*V. vinifera* cv. Pinot noir clone I-SMA 185	increased concentration of vanillic acid and decreased concentration of catechin, esculin, arbutin, astringin, pallidol, ampelopsin, D-quadrangularin and isohopeaphenol in the plants that had been inoculated by bacteria; effect of plant colonisation more prominent in the stems than in roots	[83]
Proteomic analysis of *G. diazotrophicus* PAL5 in response to plantlets	*G. diazotrophicus* PAL5	*S. officinarum* cv. SP70-1143	differential expression of 38 proteins that are associated with carbohydrates and the energy metabolism, folding, sorting, degradation processes, transcription and translation; in bacterial cells responding to the plantlets, a higher expression of the transcription elongation factor (GreA), a 60 kDa chaperonin (GroEL) and outer membrane lipoprotein (Omp16)	[61]
Proteomic analysis of *Azospirillum* spp. in response to plant root exudates	*A. brasilense* Sp7, Sp245, Sp246; *A. lipoferum* Sp59b, SpBrl7, DN64, SF50	*T. aestivum* cv. Fidel	induction of the acidic 40-kDa protein in response to root exudates	[84]
Proteomic analysis of rice roots colonised by *H. seropedicae* SmR1	*H. seropedicae* SmR1	*O. sativa* ssp. *japonica* cv. Nipponbare	bacterial response: higher level of dinitrogenase reductase NifH and glutamine synthetase GlnA; plant response: upregulation of S-adenosylmethionine synthetase, methylthioribose kinase and acireductone dioxygenase 1; stimulation of the phytosiderophores biosynthesis	[72]
Proteomic analysis of rice roots colonised by *Azoarcus* sp. BH72 and in response to ethylene and JA treatment	*Azoarcus* sp. BH72	*O. sativa* subsp. *indica* cv. IR36 and IR42	plant defence responses involving JA may contribute to restriction of endophytic colonisation in grasses; endophytic colonisation and JA induce the expression of the pathogenesis-related proteins and salt-stress induced proteins	[85]

**Table 3:** The comparative genomics studies involving endophytic bacteria.

Strain(s)	Major Findings	References
92 strains, including four endophytes: *Staphylococcus epidermidis* SE4.7, SE4.8, SE4.6 and SE2.9	identification of a distinct sub-lineage of *S. epidermidis* isolated from surface-sterilised rice seeds, which is different from the majority of human isolates; identification of the five genomic regions that are associated with rice *S. epidermidis* endophytes, i.e., encoding for methionine sulfoxide reductase (*msrA*), haloacid dehydrogenase (HAD), 4-hydroxythreonine-4-phosphate dehydrogenase (*pdxA*), repair protein (*radC*)	[89]
21 rice seed endophytes belonging to the phylum *Firmicutes*	identification of numerous genes that are related to the production of auxin and siderophores, tolerance of oxidative, heat and osmotic stresses in the endophytic strains	[90]
40 endophytes, 42 nodule-forming symbionts, 42 rhizosphere bacteria, 29 plant pathogens, 49 soil bacteria	enrichment of the endophyte-related genes: response regulator proteins CheBR and CheC, flagellum biosynthesis, motility mechanisms, transcriptional regulation of nitrogen assimilation (*nifA*), reduction of nitric oxide (*norR*), regulation of carbon storage (*sdiA*), beta-lactamase resistance (*ampR*), pyrimidine metabolism (*pyrR*), thiamine metabolism (*tenA*), glutathione peroxidase (*btuE*), glutathione S-transferase (*gst*), catalase (*katE*), nitric oxide reductase (*norR*), ATP-binding cassette (ABC), major facilitator superfamily (MFS), phosphotransferase system (PTS), solute carrier family (SLC), type IV conjugal DNA-protein transfer secretion system and nitrogenase (*nifH*)	[7]
nine endophytes: *B. phytofirmans* PsJN, *Burkholderia* spp. JK006, *A. lipoferum* 4B, *E. cloacae* ENHKU01, *Klebsiella pneumoniae* 342, *P. putida* W619, *Enterobacter* spp. 638, *Azoarcus* spp. BH72, *Serratia proteamaculans* 568	identification of the gene sets that are putatively responsible for endophytic behaviour, transporters: major facilitator superfamily (MFS) and ATP-binding cassette (ABC) transporter; secretion systems: type VI secretion system; transcriptional regulator: AraC, FrmR, AsnC, LrgB, LysR, DeoR, WrbA and two components of the winged helix transcriptional regulator proteins; plant polymer degradation: cellulases, hemicellulases, endoglucanases; detoxification: glutathione S-transferase, dehydrogenases, synthases, hydratases	[64]
three strains: *Stenotrophomonas rhizophila* DSM 14405T (endophyte), *S.* ***maltophilia*** R551-3 (endophyte), *S. maltophilia* K279 (human pathogen)	presence in the genome of *S. rhizophila* DSM 14405T of unique genes related to the endophytic lifestyle: osmotic stress protection: *cbg1*, *xynB*, *ggpS*, *ycaD*, *algJ* and type VI secretion system	[58]
six strains: *B. amyloliquefaciens* FZB42 (plant-associated bacteria), *B. subtilis* 168, *Bacillus licheniformis*, *Bacillus clausii*, *Bacillus halodurans*, *Bacillus cereus*	presence of 214 unique genes in the genome of *B. amyloliquefaciens* FZB42, among them, three encoding proteins with a collagen-related GXT structural motif, which are putatively involved in surface adhesion or biofilm formation	[91]
10 *Pseudomonas* spp. strains	diversified presence of type II, III, IV, VI secretion systems	[92]
four strains: *P. putida* KT2440 (rhizospheric bacteria), *P. putida* W619 (endophyte), *P. putida* F1 (aromatic hydrocarbon-degrading strain), *P. putida* GB-1 (manganese-oxidising strain)	presence of the *ndvB* (encoding beta-(1,2)-glucan) and two genes that are putatively involved in adhesion that encode for the autotransporter proteins (secretion type V) with a pectin/lyase/pertactin domain genes in the W619 strain that is absent in KT2440 strain	[93]
four strains: *Enterobacter* sp. 638, *S. maltophilia* R551-3, *P. putida* W619, *S. proteamaculans* 568	adaptation to utilise a broad spectrum of plant-derived compounds as a carbon source	[94]
five endophytic strains: *Azoarcus* sp. CIB, “*Aromatoleum aromaticum*” EbN1, *Azoarcus* sp. BH72; *Azoarcus* sp. KH32C; *Azoarcus toluclasticus* MF63	no unique gene cluster in *Azoarcus* sp. CIB exclusive for an endophytic lifestyle; presence of the genes that are related to the motility, adhesion, adaptation to the plant defence responses, type II, IV and VI secretion systems in *Azoarcus* spp.	[65]
four strains: *A. brasilense* CBG457 (endophyte), *A. brasilense* Sp245 (rhizospheric bacteria), *A. lipoferum* 4B (endophyte), *Azospirillum* sp. B510 (endophyte)	niche-specific presence of the genes that are related to EPS and LPS biosynthesis, adhesion, catabolic properties and plant cell wall degrading enzymes	[95]
*B. phytofirmans* PsJN and 8 other endophytic strains	presence in the genome of *B. phytofirmans* PsJN of numerous genes that are connected with the degradation of complex organic compounds and detoxification, cell surface signalling, secretion systems and quorum sensing system	[35]
*H. frisingense* GSF30^T^ and 14 other strains	lack of a type III secretion system in *H. frisingense* GSF30^T^ that is present in some related *Herbaspirillum* grass endophytes; differences in respiration, carbon, nitrogen and cell wall metabolism among *Herbaspirillum* isolates partially correlate with their different habitats	[96]
seven *Pantoea ananatis* strains	genomic differences in the genes encoding the secretion systems, putative effectors and transposase/integrases/phage-related genes	[97]
seven grapevine endophytic bacteria and 12 reference strains	lifestyle of pathogens or endophytes might be the outcome of complex, multifactorial interactions	[98]
*Kosakonia radicincitans* DSM 16,656 and 31 other strains	presence in the genome of *K. radicincitans* DSM 16656^T^ of two flagellar systems and three type VI secretion systems, which may help them to avoid pattern-triggered immunity in plants	[99]
*Pseudomonas viridiflava* CDRTc14 (endophyte) and ten other *Pseudomonas* spp. strains	presence of the genes that are related to plant-bacteria interactions: type IV pili, motility, chemotaxis, transporters, type I, II, IV, V, VI and VII secretion systems, stress response, quorum sensing and quorum quenching	[100]
*Azoarcus olearius* DQS-4 (isolated from soil, rice endophyte) and *Azoarcus* sp. BH72 (endophyte)	presence of similar genes in *A. olearius* DQS-4 and *Azoarcus* sp. BH72 that are related to plant-bacteria interactions	[101]
3837 bacterial genomes including 484 newly sequenced bacteria that had been isolated from the roots of Brassicaceae, poplar and maize	identification of 64 plant-associated protein domains that potentially mimic plant domains, enrichment in the genomes of plant-associated bacteria of the genes encoding the proteins that are related to the carbohydrate metabolism and lower number of mobile elements; in *Acidovorax* the presence of the characteristic gene families *Jekyll* and *Hyde* for non-pathogens and pathogens, respectively	[14]
108 bacterial endophytes, 56 plant bacterial pathogens, 96 rhizosphere bacteria	enrichment of the genes encoding for nitrogenase, involved in the uptake of urea cycle, transport, secretion systems and transcriptional regulation	[15]

**Table 4:** Plant mutants that have been utilised for understanding plant–endophytic bacteria interaction.

Plant	Mutant Name	Phenotype Characteristics	Bacterial Strain	Major Findings	References
*M. truncatula*	*sickle* (*skl*)	ethylene-insensitive (sickle)	*K. pneumoniae* 342	higher level of *skl* plant colonisation by bacteria	[107]
*Nicotiana attenuata*	*ir-aco1*	deficient in ethylene biosynthesis	*-*	lower bacterial diversity	[109]
*N. attenuata*	*35S-etr1*	deficient in ethylene perception	*-*	lower bacterial diversity	[109]
*A. thaliana*	*fad3/7/8*	three fatty acid desaturase genes (FAD3, FAD7 and FAD8) that are necessary to produce wild-type levels of JA	*-*	greater epiphytic bacterial diversity	[111]
*A. thaliana*	*nahG*	SA degradation, defective in defence by expressing the bacterial salicylate hydroxylase gene, *nahG*	*K. pneumoniae* 342, *S. enterica* serovar Typhimurium 14,028	no changes in colonisation by the 342 strain, a higher level of *nahG* plant colonisation by the 14,028 strain	[107]
*A. thaliana*	*nahG*	SA degradation, defective in defence by expressing the bacterial salicylate hydroxylase gene, *nahG*	*G. diazotrophicus* PAL5	higher level of colonisation of the roots and leaves of *NahG* plants by bacteria	[110]
*A. thaliana*	*npr1*	regulates the DNA binding ability of transcription factors that are involved in plant defence, disrupts the SA-mediated and SA-independent defence responses	*-*	reduced endophytic bacterial community diversity	[111]
*A. thaliana*	*npr1*	regulates the DNA binding ability of the transcription factors that are involved in plant defence, disrupts the SA-mediated and SA-independent defence responses	*K. pneumoniae* 342, *S. enterica* serovar Typhimurium 14,028	higher level of colonisation *npr1* plants by the 342 and 14,028 strains	[107]
*A. thaliana*	*sid2* (also known as *eds16*)	deficient in the accumulation of SA	*-*	reduced endophytic bacterial community diversity	[111]
*Hordeum vulgare* var. Karat	*rhl1.a*	completely hairless mutant that exhibits a disturbed pattern of root epidermis cells with indistinguishable trichoblasts	*-*	reduced complexity community	[113]
*H. vulgare* var. Dema	*rhp1.b*	develops only to the primordium stage and their tip growth is arrested after bulge forms	*-*	reduced complexity community	[113]
